# The National Association of Dental Faculties

**Published:** 1898-10

**Authors:** 


### EDITORIAL.

### The National Association of Dental Faculties.

This body met in Omaha on Friday, August 26th, with quite a full representation from the dental colleges of the country. There were but few absentees. The work done by this body was of very great importance to the profession.

There was manifested, in various ways, not only a desire but a determination that advance should be made all along the line in the matter of dental education.

A desire was shown for a more pronounced compliance with the rules and requirements of the body than ever before. A number of changes and amendments were made in the rules and regulations, all of which had in view a better condition of things than ever before existed. A very marked improvement was shown in the conduct of the affairs of some of our colleges, and it is confidently expected that the same will be observed in others, that each year will show a marked improvement, and that in the near future all will come up to a better level than has hitherto been realized.

A close observer could not be otherwise than gratified in noting what was accomplished by this meeting. As we expect to publish the proceedings in the next number of this journal, we will not here anticipate further upon what will be there shown.

